# Web-based evaluation of experts' opinions on impacted maxillary canines forced eruption using CBCT

**DOI:** 10.1590/2176-9451.20.2.090-099.oar

**Published:** 2015

**Authors:** Amirfarhang Miresmaeili, Nasrin Farhadian, Vahid Mollabashi, Faezeh Yousefi

**Affiliations:** 1 Associate professor, Hamadan University of Medical Science, School of Dentistry, Department of Orthodontics, Hamadan, Iran; 2 Assistant professor, Hamadan University of Medical Science, School of Dentistry, Department of Orthodontics, Hamadan, Iran; 3 Associate professor, Hamadan University of Medical Science, School of Dentistry, Department of Dento-maxillofacial radiology, Hamadan, Iran

**Keywords:** Cuspid, Cone-beam computed tomography, Tooth eruption, Ectopic, Internet

## Abstract

**AIM:**

This study aims at examining the difficulty in performing forced eruption of impacted maxillary canines, using CBCT information and according to experienced orthodontist's opinion. The second aim was to find the most important factors related to this decision.

**METHODS:**

Based on a careful literature review on impacted maxillary canines, ten main factors were selected to assess difficulties associated with impacted teeth. Thirty six consecutive patients with 50 impacted maxillary canines were examined and variables were measured for each impacted tooth using Dolphin 3D software. Ten orthodontists assessed the radiographs of teeth and provided their opinion on the difficulty in bringing impacted teeth into occlusion named subjective degree of difficulty (SDD). The correlation established between mean SDD of each tooth and measured variables were analyzed by means of linear regression.

**RESULTS:**

Mean SDD was 6.45 ± 1.22 for all 50 teeth. Linear regression showed a high coefficient of correlation between mean SDD and age, dilacerations, vertical height, angulation and horizontal overlap (P < 0.05).

**CONCLUSION:**

To predict the difficulty of impacted maxillary canines forced eruption, according to the opinion of experienced orthodontists, the factors age, dilaceration, angulation, overlap and vertical distance from the occlusal plane are the most important variables.

## Abstract (pt)

**OBJETIVO:**

o presente estudo tem o objetivo de avaliar, de acordo com a opinião de ortodontistas experientes, a dificuldade de se realizar a erupção forçada de caninos superiores impactados, com base em informações fornecidas por tomografia computadorizada de feixe cônico. O objetivo específico foi identificar os fatores mais importantes relacionados a essa opção de tratamento.

**MÉTODOS:**

com base em uma revisão meticulosa da literatura sobre caninos superiores impactados, selecionamos os dez principais fatores utilizados para avaliar a dificuldade em tratar dentes impactados. Trinta e seis pacientes, com um total de 50 caninos superiores impactados, foram avaliados. As variáveis para cada dente impactado foram obtidas utilizando o programa Dolphin 3D. Dez ortodontistas avaliaram as radiografias referentes aos dentes selecionados e opinaram sobre a dificuldade em alinhar dentes impactados. O grau de dificuldade foi denominado Grau de Dificuldade Subjetivo (GDS). A relação entre o valor médio de GDS de cada dente e as variáveis obtidas foi analisada por regressão linear.

**RESULTADOS:**

o valor médio de GDS foi de 6,45 ± 1,22, para todos os dentes. A regressão linear gerou um alto coeficiente de relação entre o valor médio de GDS e as variáveis idade, dilaceração, altura vertical, angulação e sobreposição horizontal (p < 0,05).

**CONCLUSÃO:**

de acordo com a opinião de ortodontistas experientes, para prever a dificuldade em se realizar a erupção forçada de caninos superiores impactados, as variáveis idade, dilaceração, angulação, sobreposição e distância vertical do plano oclusal são as mais importantes.

## INTRODUCTION

With the exception of third molars, maxillary canines are the most frequently impacted teeth, with prevalence ranging from 0.8 to 3.0%.^1^^,^ 2 ^,^ 3 Maxillary canines are considered to be important esthetically and functionally, and patients with impacted maxillary canines are found to be more difficult and time-consuming to treat than the average orthodontic patient.^4^

Localization of impacted canines can be challenging with conventional radiographic methods due to image distortion, superimposition of three-dimensional structures, image artifacts, projection errors and sometimes poor image quality.^1,5,6,7^ More recently, three-dimensional volumetric imaging systems (CBCT) have allowed more precise localization of impacted canines, using spatial relationships, with excellent tissue contrast.^5^ The costs, efficiency, and benefits of CBCT imaging are very favorable, as one single imaging session can provide many important views to locate the position of the impacted tooth.^3^

Several variables have been proposed to predict the difficulty of treating impacted maxillary canines and the likelihood of complications or failure.^8^ In a study by Fleming et al, angulation of the canine, vertical position from the occlusal plane, anterior-posterior position of the root apex and the degree of overlap of the adjacent incisor correlate with the prognosis of ectopic canines.^2^ Zucatti et al reported a strong association between the number of visits and increasing age, vertical height, and mesial displacement of the cusp tip.^8^

Canines that are angulated towards the horizontal plane, according to Pitt et al,^9^ have a poorer alignment prognosis. A buccopalatal position of the canine crown also influences treatment decisions, with palatally impacted canines being more likely to be surgically exposed, whereas those in the line with the dental arch or buccally positioned are more likely to be removed.^9^ It has been reported that the higher above the occlusal plane the canine is positioned, the poorer the prognosis for alignment.^9^ McSherry described this as "the vertical rule of thirds".^10^

Maxillary lateral incisor root resorption is the most common adverse effect associated with an impacted maxillary canine.^11^ Previous studies have shown that root resorption less than 0.60 mm in diameter and 0.30 mm in depth cannot be detected with 2D radiography.^12,13^ Alqerban^14^ found that CBCT imaging was significantly better than panoramic radiography in determining the degree of root resorption in the categories of slight and severe resorption.

Impacted teeth are notoriously more difficult to treat in adults.^15^ A study found that the success rate among patients over 30 years old was 41%, whereas the success rate for those aged between 20 and 30 years old was 100%.^8^

Presently, the prediction of impacted canines treatment success has been largely based on personal clinical experience and anecdotal evidence; therefore, a system that offers an improved assessment technique of the degree of difficulty in bringing a displaced canine into alignment will be beneficial for both patient and clinician.^9^ The related studies are mainly based on conventional radiographs, and CBCT have not been used widely for estimation of difficulty.

In the present study, the primary objective was to find the opinion of experienced orthodontists about difficulties in treating a sample of impacted canines, using CBCT information. The secondary objective was to find the main factors related to this decision.

## MATERIAL AND METHODS

There is no general agreement on the criteria used to distinguish between impacted maxillary canines that could be treated orthodontically or not. After a careful literature review on ISI website for "impacted maxillary canine", "ectopic maxillary canine", "treatment difficulty", "orthodontic treatment", "CT" and "CBCT", 237 articles were found. Among these, 11 articles were selected according to their citation and relevance.^2,4,5,6,8,9,15-19^ One expert orthodontist evaluated the articles. Age, horizontal position, vertical position, apex position, angulation, buccopalatal position and rotation factors were used in the articles with 2D radiograph.^2,4,8,9,15,16^ 3D view provides more information about impacted canines, but the studies using 3D views only evaluated incisor resorption, canine crown or root position.^5,6,17,18,19^ We thought that besides the above factors, dilaceration and transposition could also be clearly seen in CBCT scans. As a result, ten factors were selected for evaluation in this study. Table 1 provides a list of the ten factors and their related grading. Since CBCT information was not used widely in the previous articles to detect treatment difficulty of impacted canines, we decided to combine these data with expert orthodontists' opinions.

**Table 1 -:** Scales and grading proposed to establish the ten variables assessed.

Number	Variable	Value
1	Age	
	Younger than 18 years	1
	Between 18- 25 years	2
	Older than 25 years	3
2	Horizontal position in relation to adjacent teeth (overlap):	
	Cusp tip in proper normal position	0
	Cusp tip is deviated from its center, but without any overlap on lateral incisor	1
	Cusp tip have overlaped the distal half of the lateral incisor	2
	Cusp tip have overlaped the mesial half of lateral incisor	3
	Cusp tip have overlaped the distal half of the central incisor	4
	Cusp tip have overlaped the mesial half of the central incisor or passed the midline	5
3	Transposition with lateral or first premolar	
	No	0
	Yes	1
4	Vertical distance between canine tip to occlusal plan	
	Canine cusp is in proper vertical location	0
	Canine cusp is in the coronal region	1
	Cusp tip lies in the cervical third of the incisor root	2
	Cusp tip lies in the middle third of the incisor root	3
	Cusp tip lies in the apical third of the incisor root	4
	Cusp tip is supra-apical to the incisor root	5
5	Apex location	
	Canine root is in proper normal location	0
	Canine root is deviated from its center, but without any overlap on first premolar	1
	Canine root is in the mesial half of the first premolar	2
	Canine root is in the distal half of first premolar	3
	Canine root is in the mesial half of the second premolar	4
	Canine root is distal to the midline of the second premolar	5
6	Angulation in relation to the occlusal plan	
	Angle ≤ 30 degrees	4
	Angle between 30-45 degrees	3
	Angle between 45-60 degrees	2
	Angle above 60 degrees	1
7	Root dilacerations	
	No	0
	Yes	1
8	Incisor root resorption	
	No resorption	0
	Slight resorption when less than midway between pulp canal and cementum is resorbed	1
	Moderate resorption when more than midway between pulp canal and cementum is resorbed	2
	Severe resorption when the pulp is exposed	3
9	Buccopalatal position	
	Canine located in the middle of alveolar bone	1
	Canine located in the buccal surface of alveolar bone	2
	Canine located in the palatal surface of alveolar bone	2
10	Rotation: (in 3D view)	
	No	0
	Yes	1

These ten factors all have different scales of measurements and a different range of ratings depending on the nature and importance of each factor which were generally based on the reviewed literature. With the exception of age, all other factors were measured on the CBCT scan. Because CBCT provided a great amount of information for each patient, we decided to use a more structured format so that we could analyze tooth location and surrounding structures in a smooth and convenient way. Each factor was determined using the following sequence in CBCT: 

## » Step one: frontal view

**Figure 1 -:**
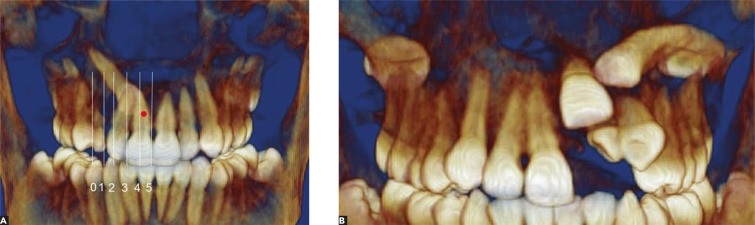
Four-step evaluation. Step one: Reconstructed 3D image in frontal view used to examine impacted canine. A) Overlap and B) Transposition.

A) The horizontal position of the impacted tooth was evaluated in relation to adjacent incisors (Fig 1A).

B) Transposition was evaluated, if present (Fig 1B).

## » Step two: lateral view

(Right and left side depending on the location of the impacted tooth).

**Figure 2 -:**
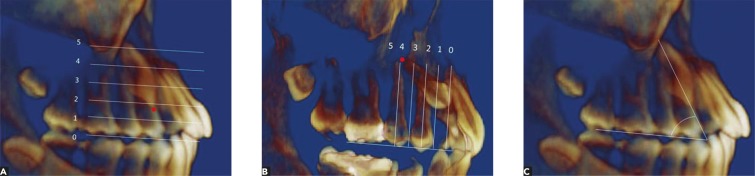
Four step evaluation; Step two: Reconstructed 3D image from sagittal view to examine impacted canine A) Vertical position B) Apex location and C) Angulation in relation to occlusal plan.

A) The vertical position of canine tip was measured in relation to adjacent teeth (Fig 2A).

B) The apex position of the impacted tooth was recorded in relation to adjacent teeth (Fig 2B).

C) The angulation of the impacted tooth was calculated in relation to the occlusal plane (Fig 2C).

## » Step three: axial view

**Figure 3 -:**
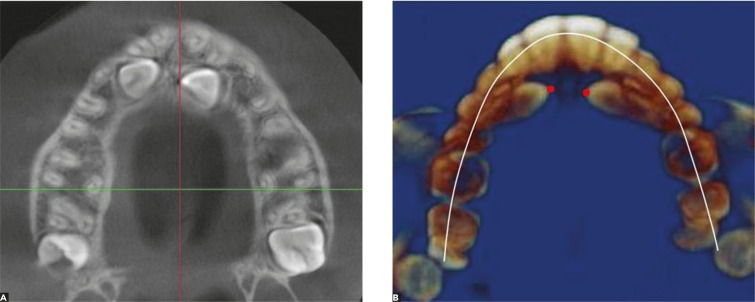
Four-step evaluation. Step three: Axial view to examine impacted canine. A) Induced root resorption at the closest contact with incisor root and B) Buccopalatal position at the crown level.

A) The extent of possible incisor root resorption was measured at the location where the canine tip was closest to incisor root (according to the distance between pulp and cementum) (Fig 3A).

B) The buccopalatal position of the impacted tooth was determined in relation to the center of the dental arch (Fig 3B).

## » Step Four

A) Dilacerations and their location were recorded.

B) Rotations were recorded (mesial and distal 3D views of canine crown were determined and its angulation with the line of arch circumference was measured. If this angle was zero, the teeth had no rotation).

A sample of 36 patients with 50 impacted maxillary canines were collected and had CBCT scans taken by a Newtom 3G device (Quantitative Radiology, Verona, Italy), with minimum slice thickness of 0.4 mm, in which the maxilla and impacted canines could be seen completely. To measure these factors, we imported the DICOM files into Dolphin 3D software designed for analysis of CBCT data. Subsequently, we adjusted the orientation and used the transparency tool to increase image clarity so that any impacted teeth could be easily seen. Then, each factor was measured on Dolphin 3D.

**Figure 4 -:**
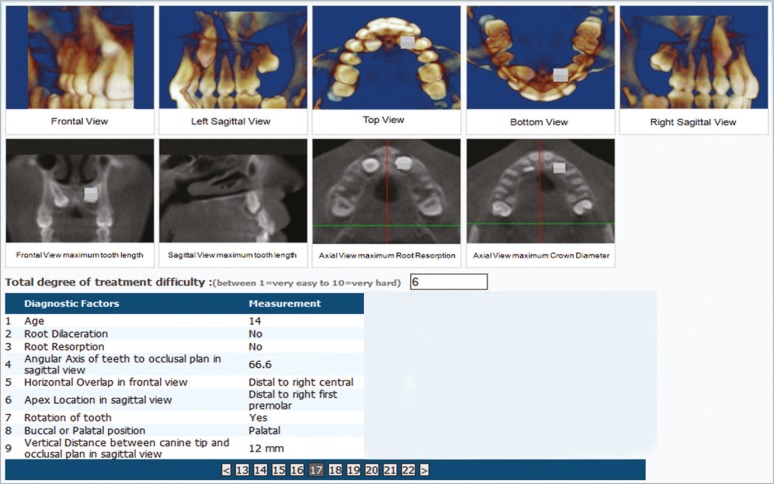
Web-base questionnaire for 50 impacted canines that each expert orthodontist evaluated for subjective degree of difficulty. Measurements of all variables for each tooth were recorded.

According to our second aim, we planned to assess the difficulty of impacted maxillary canine forced eruption to the occlusal level using the opinion of a group of experienced orthodontists. To facilitate our four-step examination, for this phase, we prepared four 2D and five 3D images for each impacted tooth to be uploaded easily in a website devoted to this study (www.canineimpaction.com shown in Fig 4). We sent a participation request to 20 well-known national and international orthodontists.

After examining the records of each patient, the evaluators were asked to suggest a grade for the difficulty in aligning, or bringing into occlusion, impacted canines. This was done by allocating a score based on a scale from 1 to 10, with 1 being very easy and 10 extremely difficult. The evaluators' score for each tooth was termed subjective degree of difficulty (SDD).

Following data collection, correlation between mean SDD and the measured variables was established by means of linear regression in SPSS 16.

## RESULTS

Thirty six consecutive patients (29 females and 7 males), with 50 impacted maxillary canines, referred to the orthodontic clinic of the School of Dentistry for orthodontic treatment, were included in this study. Fourteen patients had bilateral impaction. Nine canine impactions were on the right, while 13 were on the left side. The subjects ranged in age from 12 to 34 years old (mean 19.08 ± 5.8 years).

The main observer examined all impacted maxillary canines and measured each one of the ten factors. Distribution of percentages for each factor can be seen in Table 2.

**Table 2 -:** Distribution percentage of diagnostic factors in 50 impacted canines.

Number	Variable	Value
1	Age	
	Younger than 18 years	52%
	Between 18- 25 years	34%
	Older than 25 years	14%
2	Horizontal position in relation to adjacent teeth (overlap)	
	Cusp tip in proper normal position	12%
	Cusp tip is deviated from its center, but without any overlap on lateral incisor	2%
	Cusp tip have overlaped the distal half of the lateral incisor	14%
	Cusp tip have overlaped the mesial half of lateral incisor	18%
	Cusp tip have overlaped the distal half of the central incisor	26%
	Cusp tip have overlaped the mesial half of the central incisor or passed the midline	28%
3	Transposition with lateral or first premolar	
	No	100%
	Yes	--
4	Vertical distance between canine tip to occlusal plan	
	Canine cusp is in proper vertical location	--
	Canine cusp is in the coronal region	--
	Cusp tip lies in the cervical third of the incisor root	40%
	Cusp tip lies in the middle third of the incisor root	42%
	Cusp tip lies in the apical third of the incisor root	12%
	Cusp tip is supra-apical to the incisor root	6%
5	Apex location	
	Canine root is in proper normal location	--
	Canine root is deviated from its center, but without any overlap on first premolar	--
	Canine root is in the mesial half of the first premolar	4%
	Canine root is in the distal half of first premolar	6%
	Canine root is in the mesial half of the second premolar	36%
	Canine root is distal to the midline of the second premolar	54%
6	Angulation in relation to the occlusal plan	
	Angle ≤ 30 degrees	14%
	Angle between 30-45 degrees	30%
	Angle between 45-60 degrees	38%
	Angle above 60 degrees	18%
7	Root dilacerations	
	No	54%
	Yes	46%
8	Incisor root resorption	
	No resorption	12%
	Slight resorption when less than midway between pulp canal and cementum is resorbed	88%
	Moderate resorption when more than midway between pulp canal and cementum is resorbed	--
	Severe resorption when the pulp is exposed	--
9	Buccopalatal position	
	Canine located in the middle of alveolar bone	20%
	Canine located in the buccal surface of alveolar bone	8%
	Canine located in the palatal surface of alveolar bone	72%
10	Rotation: (in 3D view)	
	No	20%
	Yes	80%

**Figure 5 -:**
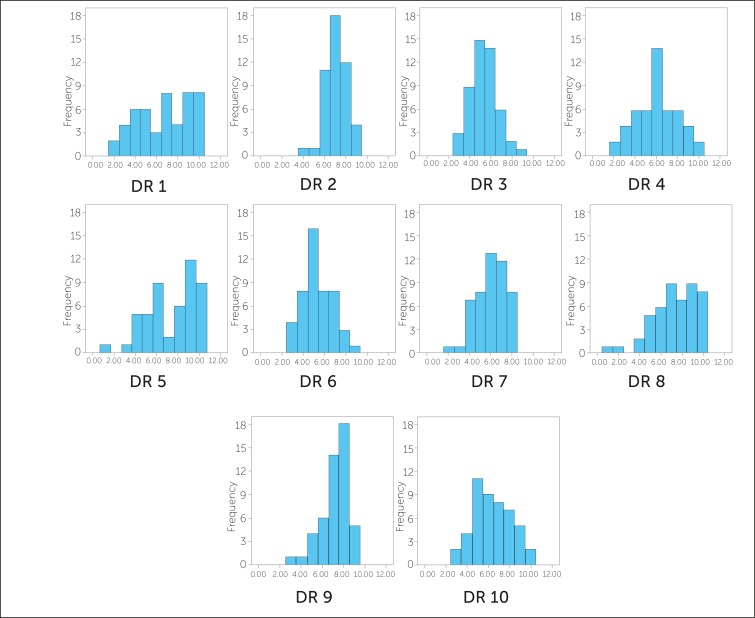
Histograms of subjective degree of difficulty (SDD) of the whole sample derived from each one of the ten evaluators

**Figure 6 -:**
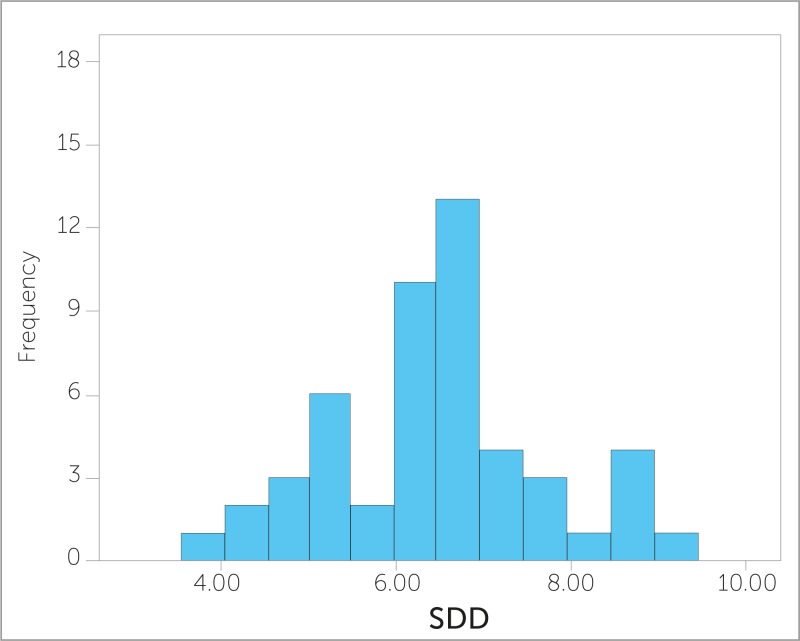
Histogram of mean subjective degree of difficulty (SDD) for each impacted canine specified by all evaluators.

Ten orthodontists accepted to participate in the study and had an average number of years in practice of 22.7 ± 12.02 years. The frequencies of SDD by each evaluator are shown in Figure 5. Mean SDD was computed by ten evaluators for each tooth (Fig 6) and its total mean was 6.45 ± 1.22 for all impacted teeth.

Simple linear regression was used to assess the correlation between mean SDD and nine factors. The effective variables entered the model at P = 0.001 and were excluded at P = 0.05 in backward stepwise by SPSS 16.

Results show that age, dilacerations, angulation, overlap and vertical distance had significant correlation with mean of SDD (Table 3).

**Table 3 -:** Simple linear regression shows five factors with significant correlation with mean SDD.

Model	B	Standard error	B	t	Sig.
Constant	1.327	0.458	---	2.898	0.006
Age	0.402	0.126	0.237	3.179	0.003
Dilaceration	0.546	0.175	0.224	3.122	0.003
Angulation	0.352	0.097	0.271	3.617	0.001
Overlap	0.374	0.054	0.496	6.913	0.000
Vertical distance	0.760	0.109	0.535	6.957	0.000

## DISCUSSION

When orthodontists consider the possibility of treating an impacted canine, they are facing several problems. The first is the potential duration of treatment, if guided eruption is considered. Should that be the case, the tooth is brought into occlusion by a combination of surgical and orthodontic interventions. The second problem is the esthetic and functional results of treatment, which may not fulfill patients' best interests. The third problem is the risk of damage to neighboring teeth during treatment, especially in cases in which surgical exposure is needed. It would be of great benefit to both the patient and the clinician if the orthodontist, before treatment, had a better idea of treatment difficulties involved in treating an impacted tooth. Becker et al^16^ concluded that the major reasons for failure of impacted canine force eruption were inadequate anchorage, mistaken location, directional traction and ankylosis.

It could be easier for the clinician to determine whether to extract an impacted tooth or attempt to force erupt it if a systematic approach were available. A treatment difficulty index has been proposed according to conventional radiographs,^9^ but rare studies^6^ are available using CBCT to assess treatment difficulty. Bjerklin and Ericson^18^ found that treatment planning of 43.7% os patients were modified when new information gleaned from computed tomography were presented to the examiner.

CBCT provides detailed information about impacted teeth. It is important that this information be organized so as to prevent confusion and reduce the time needed for evaluation in the diagnosis and treatment planning process. The evaluation of each one of the ten factors was structured in a four-step approach, so that the clinician could view impacted teeth conveniently in a simple protocol, in addition to measuring each factor systematically.

Kau CH^5^ introduced a new measuring scale, in cases of impacted canines, based on three different viewpoints of CBCT, in order to grade the difficulty of impaction and the potential efficacy of treatment. The author believed that the sum of scores for the cusp tip and root tip in the three views determined the anticipated difficulty of treatment. This grading may be useful in treatment planning, but its clinical usefulness and reliability have not yet been evaluated.

In our study, the most important factors listed in published articles about canine impaction and their possible role with respect to treatment difficulty were measured and provided for the evaluators. Comparison of our experts' opinion and variables in each patient showed that age, dilaceration, angulation, overlap and vertical distance from the occlusal plane have significance correlation with SDD. In a study by Fleming et al,^2^ angulation of the canine, vertical position from the occlusal plane, anterior-posterior position of the root apex and the degree of overlap of the adjacent incisor correlate with the prognosis of ectopic canines. Zucatti et al^8^ reported a strong association between the number of visits and increasing age, vertical height, and mesial displacement of the cusp tip. Pitt et al^9^ proposed a treatment difficulty index according to horizontal position, age, vertical height, buccopalatal position, rotation, midline, angulation and alignment. These results showed that a combination of five major variables out of ten could be used to predict difficulty based on a pool of experts' opinion.

Although examination of CBCT scans based on a full viewer version may be ideal for experts evaluation, we arranged the evaluation using nine picture format due to unavailability of Dolphin 3D viewer at that time and the time needed to assess 50 impacted teeth. This makes web-based questionnaires more convenient to upload smaller size data files. The kind of questionnaire used in this study helps us show images of impacted teeth in 3D format and provides more convenience for evaluation all around of the world. 

To assess impacted canine treatment difficulty, the number of evaluators and their clinical experience are of utmost importance. Botticelli et al,^6^ in her study, used the opinion of eight dentists to compare 2D *versus* 3D imaging used for diagnosis of unerupted maxillary canines, but only three of them had more than five years of experience. Bjerklin^18^ counted on only one examiner to assess 80 patients' records. In another study, he sent a questionnaire of three patients to 182 orthodontists from Sweden, with at least one year of experience, to assess CT scans for resorption, but not 3D localization.^17^ In the present study, we used CBCT scans of 50 impacted teeth for 3D localization and resorption according to opinion of ten expert orthodontists with at least 10 years experience.

CBCT per se could not be a perfect tool to assess treatment difficulty, as there must be other important diagnostic criteria, such as patient's preferences, functional problems, soft tissue drape etc, which must be included in a diagnostic setup. An outcome analysis based on esthetics, periodontal conditions, occlusal function of impacted tooth and treatment follow-up,^20^ in addition to radiographic analysis, would be the gold standard of decision making.

## CONCLUSION

To predict the difficulty of impacted maxillary canines forced eruption, according to the opinion of experienced orthodontists, age, dilaceration, angulation, overlap and vertical distance from the occlusal plane are the most important variables.
