# Erratum

**DOI:** 10.1590/2176-9451.20.4.016-016.err

**Published:** 2015

In the article Web-based evaluation of experts' opinions on impacted maxillary canines forced eruption using CBCT, published at Dental Press Journal of Orthodontics, 20(2):90-99, on page 90, where it was written:

Amirfarhang Miresmaeili^1^, Nasrin Farhadian^1^, Vahid Mollabashi^2^, Faezeh Yousefi^3^

^1^Associate professor, Hamadan University of Medical Science, School of Dentistry, Department of Orthodontics, Hamadan, Iran.

^2^Assistant professor, Hamadan University of Medical Science, School of Dentistry, Department of Orthodontics, Hamadan, Iran.

^3^Associate professor, Hamadan University of Medical Science, School of Dentistry, Department of Dento-maxillofacial radiology, Hamadan, Iran.

It should be written:

Amirfarhang Miresmaeili^1^, Ib Leth Neilsen^2^, Masoud Varshosaz^3^, Nasrin Farhadian^4^, Vahid Mollabashi^5^, Abbas Moghymbeigi^6^, Faezeh Yousefi^7^

^1^Associate professor of Orthodontics department, School of Dentistry, Hamadan University of Medical Science, Hamadan, Iran.

^2^Professor of Orthodontics, University of California, San Francisco, USA.

^3^Assistant Professor of Dentomaxillofacial Radiology department, School of Dentistry, Shahid Beheshti University of Medical Sciences, Tehran, Iran.

^4^Associate professor of Orthodontics department, School of Dentistry, Hamadan University of Medical Science, Hamadan, Iran.

^5^Assistant professor of Orthodontics department, School of Dentistry, Hamadan University of Medical Science, Hamadan, Iran.

^6^Associate professor of Biostatistics and Epidemiology department, School of Public Health, Hamadan University of Medical Sciences, Hamadan, Iran.

^7^Assistant professor of Dentomaxillofacial Radiology department, School of Dentistry, Hamadan University of Medical Science, Hamadan, Iran.

